# Family Orchards and Health-Related Quality of Life in the Elderly. A Protocol for a Study in Las Hurdes (Spain) Based on an Ethnographic Approach

**DOI:** 10.3390/ijerph18031059

**Published:** 2021-01-25

**Authors:** Miguel Madruga, Jorge Carlos-Vivas, María Mendoza-Muñoz, José Carmelo Adsuar, Lorenzo Mariano-Juárez, David Conde-Caballero

**Affiliations:** 1 Department of Didactics of Musical, Plastic and Body Expression, Faculty of Sport Sciences, University of Extremadura, 10071 Cáceres, Spain; 2 Promoting a Healthy Society (PHeSo) Research Group, Faculty of Sport Sciences, University of Extremadura, 10071 Cáceres, Spain; jorge.carlosvivas@gmail.com (J.C.-V.); mamendozam@unex.es (M.M.-M.); jadssal@unex.es (J.C.A.); 3 Department of Nursing, School of Nursing and Occupational Therapy, Interdisciplinary Study Group on Society, Culture and Health (GISCSA), University of Extremadura, 10003 Cáceres, Spain; lorenmariano@unex.es (L.M.-J.); dcondecab@unex.es (D.C.-C.)

**Keywords:** ethnography, aging, health, family orchards, quality of life, study protocol, rural areas

## Abstract

Demographic evolution is resulting on an aged population increment in Spain. This growth has been more relevant in rural areas, where the population has traditionally lived under hard socio-economic conditions and leveraging the natural resources such as food from family orchards to survive. Studies that have investigated the possibilities and uses of these traditional family orchards today in relation to health-related quality of life in the elderly are scarce. Based on a previous ethnography, this mixed research aims to describe a protocol that will evaluate the effects of the use of traditional family orchards as a daily resource on fitness and quality of life of the elderly population in Las Hurdes (Spain). Body composition, fitness, mental health, health-related quality of life, and activity-related behaviors of participants will be assessed. The outcomes of this study might enable us to design further tailored physical exercise-based interventions using family orchards as an adequate resource to improve the health-related quality of life and fitness of the elderly in rural areas. In addition, the study detailed here might also be applied to other similar rural areas in Spain and worldwide.

## 1. Background

Population aging is a challenge to modern societies [1,2]. Every country in the world is experiencing an increase in the number and proportion of elderly people in its population [3]. This situation is due to different causes, including improvement in the quality of life and access to health resources that result in lower mortality in old age, and a significant decrease in the birth rate [4]. The phenomenon falls within what has been called “the second demographic transition” [5]. In this context, in Spain, according to the National Statistics Institute (INE), the Ageing Index (AI) has increased from 37.35 to 120.46 in the last 30 years (1978–2018), with a percentage of older people over the total population of 19.1%, one of the largest in the world [6]. Projections put this percentage at 29.4% in 2068 [7]. Moreover, the impact of this aging is greater in the rural areas where, since the 1960s, internal migratory movements have determined a negative demographic balance [8]. Thus, there are some regions in Spain where a very old population is living without support of younger people, together with the usual problems in rural areas such as more precarious social and health services.

**Figure 1:**
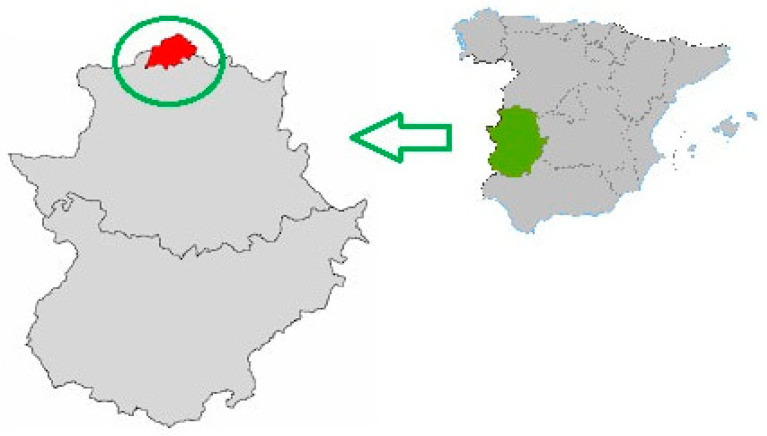
Situation of the region of Las Hurdes in the autonomous community of Extremadura (Spain).

The Spanish autonomous community of Extremadura is a paradigm in this sense. Located in the southwest of the country, which borders Portugal (Figure 1), it is a region with a very old population (AI: 140.84) [9] that is also historically poorly communicated, sparsely populated, and impoverished, with a fragile economy. Within this region, the area of Las Hurdes is a particularly sensitive area due to its social and geographical conditions. Located in the northwest of Extremadura, it has been considered as a place that is outside of history [10] (p. 42). It has been the paradigmatic expression of misery in Spain for a long time, representing hunger and the most extreme poverty, that has impacted heavily on the inhabitants’ fitness and quality of life [11]. The diet of this population was essentially based on the only foods available, which were mainly produced in their own family orchards, essential for the economy of the area throughout its history [12]. Given this past, today the zone presents a very old population, with villages like Casares de las Hurdes, representing an extreme situation, with 55.4% of people over 65 years of age in 2019 [6].

In this context, the consequences of the combination of an aging rural population, depopulation, precarious health and social services, and an economically depressed area where hunger was present until a few years ago—about the 1950s—has led the population to suffer hard socio-economic conditions over the last 50 years; thus, health-related problems could be expected nowadays in the elderly. A group of anthropologists has been carrying out some field work in this area for two years, doing qualitative research linked to the traditional tools of ethnographic fieldwork interested in the health conditions and perceptions of the elderly [13]. When we asked non-dependent old persons about their “perception of health” or “quality of life”, most of the answers were positive, and when we asked for the reasons, most of the answers referred to physical activity: “Here we’re fine”; was the most common answer to our questions, even if the questions were asked to very old people. In this sense, the relationship between health perception/quality of life and physical exercise is widely accepted in the scientific literature [14,15]. However, what kind of physical exercise do the aging people do in an area like this without social services?

Routinely the answers to this question referred to the activities carried out in the family orchards, the same orchards that have been used to obtain food and survive in the area throughout all their lives: “We are fine because we move a lot in the orchards!” Family orchards are defined as a traditional agricultural production system managed with family labor [16]. In addition, the owners usually maintain the family orchards, as they received them from their parents because they represent an ethnological heritage of the first order with traditional knowledge [17], passed down from generation to generation, from parent to children [18]. Moreover, they are sustainable and small agroecosystems developed over generations [19], where ecological, agronomic, cultural, social, and physical processes take place [20,21]. As we have already mentioned, this type of family orchards has traditionally been the only mechanism that has enabled low-income families to overcome the socio-economic problems in the area of Las Hurdes, since the family tended to take advantage of the resources obtained from the land. So, they have been an essential support for the region’s economy, to the point that, as indicates Catani [22], “the only thing that feels comfortable to a “Hurdano” is to stay near his family orchard.”

However, at present, these small pieces of land, which are part of the heritage, have important implications not only to provide the population with food—and therefore sustainability—but also in affecting their health and quality of life as the elderly people have confirmed. Today, the role of these natural spaces includes leisure and occupational aspects that may have a positive influence on the mental health of the elderly people, as well as encouraging physical activity and sustainability of a distinctly aging population.

Previous scientific literature shows that tasks performed in the family orchards may have a positive impact on older adults’ behaviors, well-being, and quality of life, including decreased falls and a reduction in the use of psychotropic medications, improved memory, enhanced social interaction, the achievement of meaningful goals, and enhanced interpersonal intimacy [23,24,25,26]. However, there is a lack of research that analyzes the specific effects of work in the orchards on the fitness and quality of life of elderly people in rural environments. Previous research has shown that similar activities such as gardening could be classified as low-to-moderate intensity physical activity, which require whole-body exercise, which helps owners to maintain a healthy fitness level [27]. De Keijzer et al. (2019) informed that the proximity to natural green and blue areas is related to slower decreases in walking speed and grip strength in people aged between 50 and 74 years from urban areas [28]. Other studies showed that living near natural environments increases physical activity levels [29,30,31], decreases psychological stress and improves perceived health among their dwellers [32,33,34,35]. Moreover, participation in agricultural activities leads people to increase their physical activity level, performing both fine motor activities (such as cutting or grafting) and gross motor activities (such as digging or ploughing) [36,37] and an association has been found between the implementation of gardening and a reduced risk of obesity and cardiovascular disease [36]. Therefore, scientific evidence tends to show that natural environments and family orchards may impact on health status, fitness and daily life in rural dwellers. However, the complexity of rural nature and the daily living conditions cannot be easily synthesized [38], considering their rooted features, habits, past conditions and traditions, and other context parameters that should be considered at different levels.

For all these reasons, from an anthropological ethnographical viewpoint, a second line of investigation has been derived, trying to discover to what extent family orchards are positive elements for the health of the elderly in Las Hurdes. To answer this question, we decided to create a multidisciplinary working group between anthropologist and specialists in Sports Sciences to do mixed research. The joint multidisciplinary study aspires to link previous research with such contemporary topics as health. Thus, we present here a study protocol which will evaluate the effects on health and quality of life of the use of traditional family orchards as a daily resource for the elderly of Las Hurdes. The results, in the case that they are positive, could be very interesting, since it is a very easy activity to encourage in other rural areas.

## 2. Materials and Methods

### 2.1. Study Design

**Figure 2:**
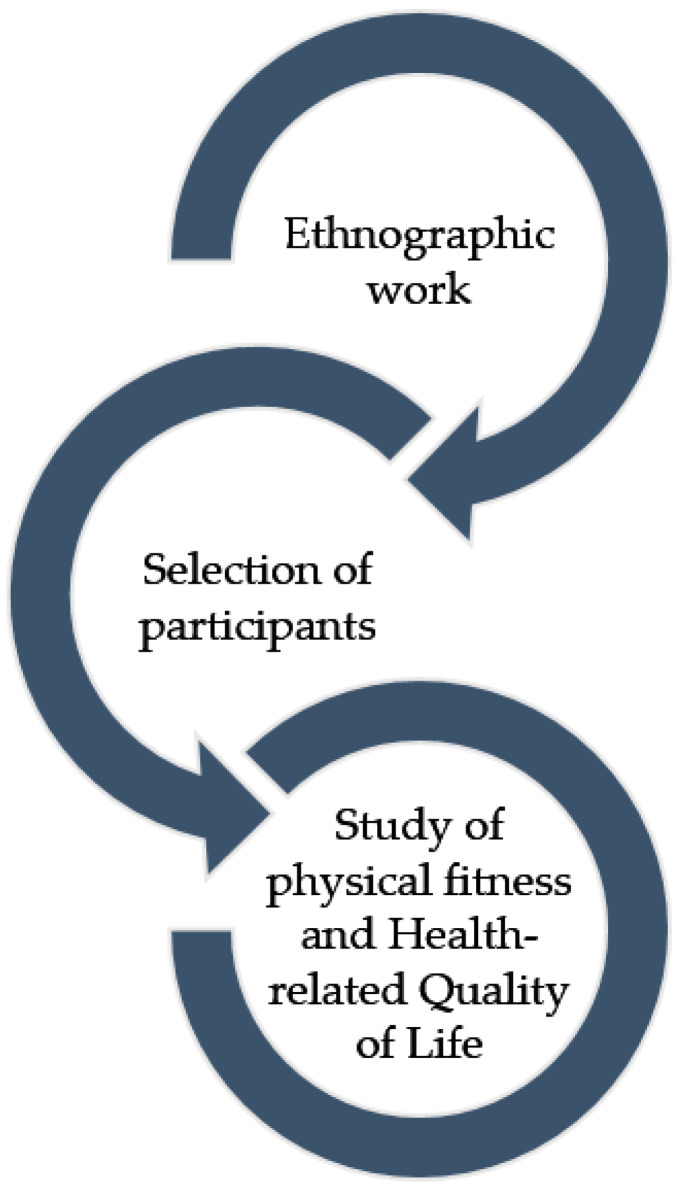
Study protocol Implementation Sequence.

This research is based on a previous ethnographic work that allowed us to know the potentiality of this approach and to have a good access to the object of study. Based on this information, a new and more targeted ethnography will allow us to obtain the sample of informants and participants in order to carry out a mixed-type research. From there a qualitative study (phase 1) and a quantitative study will be carried out (phase 2). Semi-structured interviews, observations and a field-diary will be used to collect qualitative data during the phase 1 and body composition, fitness, mental status, health-related quality of life, and activity behaviors will be assessed using different validated instruments during the phase 2. The study protocol sequence is shown in Figure 2.

### 2.2. Ethics Approval

The present research was approved by Bioethics and Biosafety Committee at the University of Extremadura (approval number: 61/2020. Date 13 July 2020).

### 2.3. Participants

To make the sample calculation, the Visual Analogic Scale (VAS) of the EQ-5D-5L has been taken as a reference. Accepting an alpha risk of 0.05 and a beta risk of 0.2 in a two-sided test, 22 subjects are necessary in first group and 22 in the second to recognize as statistically significant a difference greater than or equal to 16.44 units [39]. The common standard deviation is assumed to be 18.43 [40].

**Figure 3:**
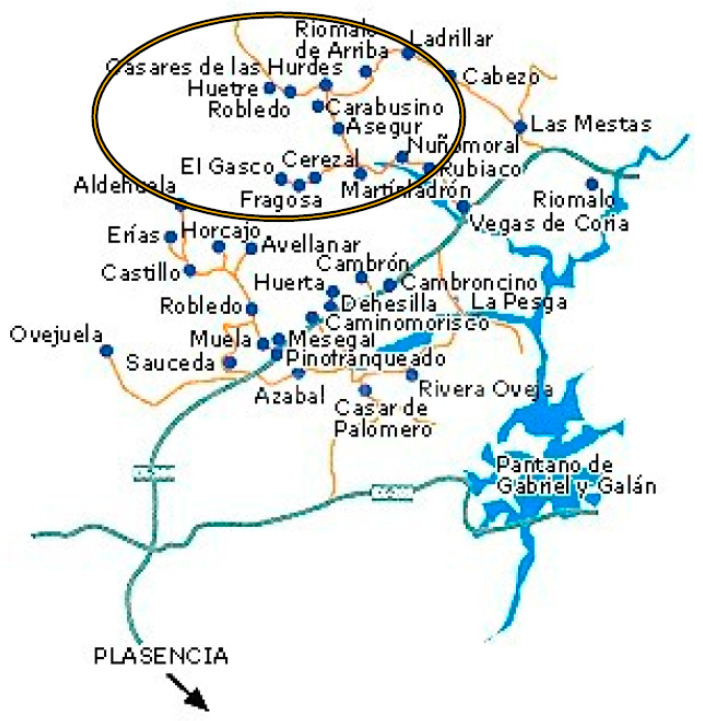
Planned fieldwork area for the project.

A purposeful sampling design will be used to systematically represent a variety of perspectives on the topic under study [41]. Participant recruitment will take place in the selected geographic area marked on Figure 3 where there is a higher concentration of family orchards. Based on previous experiences, Casares de las Hurdes and its surroundings will be the preferred area since the previously developed work will provide an easier access to informers and participants. A total of 44 participants will be recruited from the ethnographic fieldwork and assigned to two groups (22 with family orchards and 22 without family orchards). We believe that it could be a representative sample size as the study will be conducted in a depopulated environment, since several rural and depopulated contexts exist in other areas of Spain. The 44 participants selected according to the inclusion criteria will be part of both phase 1 and phase 2 of the study.

To be included in the sample with family orchards, participants will must comply the following eligibility criteria for both stages:

- Age > 65 years old.
- To be living at present and during the last 12 months in Las Hurdes, Extremadura, Spain.
- Not to be institutionalized (i.e., not to be subject to the living arrangements of institutions such as nursing homes, day centers, or care homes).
- Not to suffer any cognitive impairment considering the Mini-Mental State Examination score. This is a 30-point test whose thresholds for cognitive impairments are ≤9 points, “severe”; 10–18 points, “moderate”; 19–23 points, “mild”; and ≥24 points, “normal cognition” [42]. The test will be applied during semi-structured interviews. If at this time the score is not sufficient, the participant will be released for both phases of the study.
- Not to present health contraindications or medical conditions such as suffer from severe cardiovascular risk or severe back injuries that prevent performing the assessment tests. Participants will be required to present a health certificate from their primary health physician.

For the second stage of the study, participants will additionally need to meet the following inclusion criteria:

- To carry out activity in family gardens frequently, at least three times a week.
- Not to engage in any significant physical activity other than tending family orchards.
- To have accepted voluntary participation in the study and signed an informed consent.

To be considered into the sample without orchards, participants had to comply the same eligibility criteria as previously described, except to frequently carrying out activity in family orchards.

### 2.4. Data Collection and Measures

Based on knowledge of the field from a previous work, we will carry out a specific ethnography to select the study participants. Data and empirical materials regarding the ethnographic fieldwork (phase 1) will be collected through in-depth semi-structured interviews, and participant observation in the field. A field diary will allow to build up a more comprehensive image of subjects through providing additional information on specific contexts and issues [43,44]. All research techniques are shown in Table 1.

#### 2.4.1. Semi-Structured Interviews

Semi-structured interviews will be conducted with informers, meaning that predesignated core topics, but not specific questions, will be covered in each session, to allow the elaboration of areas whose significance emerges as the interviews proceed. This approach will ensure that the specific areas of interest will be covered and allow unanticipated topics to emerge. Core topics for interviews will include (1) well-being and health perception; (2) quality of life sense; and (3) type of activity in traditional orchards. Privacy will be protected by conducting the interviews in locations where the conversation cannot be overheard. Interviews will be usually conducted in the participant’s own native language, probably in Spanish. Interviews will be audio and video-recorded with the previous consent and permission of the interviewee and have an average length of about 40 min, considering that these are older people who may feel tired during the interview.

Participants will be asked about some specific points during the first session: age, income, pathology presence, educational level, marital status, and different questions about orchard management (e.g., growing type, daily and weekly time dedicated to orchard management, type of activity, covered distance during a working day, means of transport to go to the orchard, etc.).

#### 2.4.2. Participant Observation

Observations are a hallmark of ethnographic data collection that involve the presence of the researcher in the naturalistic environment to record events and activities that could be interesting to the research inquiry [45]. Participant observation will allow the researcher to have a direct view of events under study to complement the information collected in the interviews. The following activities will be observed by researchers in the focus area: (1) local people’s daily routine; (2) type of physical activity carried out in the family orchards; and (3) daily activities in line with the sense of well-being. Participant observation sessions will last throughout all the field works in the area.

#### 2.4.3. Data Preparation and Analysis

Once the data collection procedures are finished, the ethnographic fieldwork information will be prepared and analyzed. The Dédalo Platform that is specialized in management of the Intangible Cultural Heritage and oral sources will be used to ensure the correct use and treatment of the obtained information, since we are aware that the collected information belongs to the interviewees. Dédalo Platform Intagible Heritage Management is a web-format digital private access repository, where the informers’ testimonies will rest in the “cloud”, both their original video/audio and transcript formats. Likewise, the obtained information from questionnaires will be processed through specific office packages for this purpose.

#### 2.4.4. Physical Fitness and Health-Related Quality of Life

Considering this field work and the selection of participants who met the specific inclusion criteria, the second research line of the project will be developed (Phase 2). It will assess the impact that the use of orchards currently has on physical activity levels and health-related quality of life of participants. The following assessment will be conducted:

**Balance**

“Time Up and Go (TUG) test.” After getting up from a chair, participants will walk in a straight line a distance of 2.40 m [46], 3 m [47], and 10 m [48] (depending on the variant of the test), make a 180° turn and return to the chair to sit on it again. The time will start at the “go” signal and stop when the participant touches the back of the chair with his/her back. Each test will be performed twice, with a one-minute recovery time between each test, and a familiarization test will be performed before recording these two measurements. The best attempt will be considered for each variant.

“One-leg stance.” Participants will be timed to one-leg balance with crossed-arms over their chest. The time will count from when the foot is lifted off the ground and will end when: (1) the arms are not crossed, (2) lifted foot touches the ground, (3) movement of supported foot, and (4) maintain position over than 45 s. The assessment will be performed both open and close eyes, and in the latter case, the time will also end when the eyes are opened. Three attempts will be made with each leg both with eyes open and closed and the best value will be recorded in each case [49,50].

**Fear of Falling**

The Falls Effectiveness Scale-International (FES-I) questionnaire will be used to assess the fear of falling, which has been validated by the Fall Prevention Network Europe (ProFaNE) and is a widely accepted tool to assess the fear of falling [51]. Furthermore, it is valid and reliable in different languages [52], and its Spanish version will be used for this study [53]. This questionnaire evaluates the concern about falls in a series of activities of daily life and consists of 16 items with a four-point scale (1 = not very concerned to 4 = very concerned); that is, 16 would be the best possible value and 64 the worst. The fear of falling will also be assessed through the Visual Analogical Scale for Fear of Falling (VAS-FOF) [54].

**Body Composition**

A stadiometer (Seca 22, Hamburg, Germany) will be used for measuring height and weight. Waist and hip circumferences will be also recorded using an anthropometric tape (Harpenden Anthropometric Tape, Holtain Ltd., Crymych, United Kingdom) and with participants on a standing position. In addition, the body composition will be evaluated with a bioimpedance meter (TANITA MC 780MA).

**Physical condition**

“The 6-min walk test.” Participants must walk a 45.7 m rectangular course for 6 min. The maximum distance walked by each participant will be recorded [47].

“Lower body strength.” The 30-s standing test will measure how many times participants can, from a sitting position with their back supported, sit on and get up from a chair for 30 s without arm propulsion [55].

“Upper body strength.” This test will measure how many times participants are able to lift a weight of 2.3 kg in their hand by flexing their arm for 30 s [55]. A grip test with a digital dynamometer will also be performed (TKK 5101 Grip-D; Takey, Tokyo, Japan) [56]. Two repetitions will be performed with each hand alternately. The average of both attempts of each hand will be used for the analysis.

“Muscular strength and resistance of the trunk.” For this evaluation a test of abdominal and trunk muscular resistance will be carried out. To assess the strength of the trunk flexor muscles, participants will be asked to lie supine on a mat and to rise with 90 degrees of flexion on the hip and knee. To assess the strength of the extensor muscles, participants will lie prone and lift the shoulders off the floor by bending or extending the trunk. Both positions must be kept as long as possible, not exceeding 5 min [57].

“Upper-Limb Flexibility.” This will be evaluated by the back-scratch test, which measures the full shoulders range of motion of the shoulder. It consists of determining the distance between (or overlap of) middle hand fingers behind the back using a ruler or tape. Two attempts will be made and the best of both will be registered for both arms. The average of the two previous measurements will be used for analysis [55].

“Flexibility of the lower extremities.” The sit and reach test will be used to evaluate this parameter. Participants will sit with one leg extended and lean forward by sliding their hands across their leg until touching or overpassing their toes. The measurement will be taken in cm [58]. Two attempts will be made for each leg and the best value will be recorded for each one. For the analysis, the best measurement of each leg will be averaged.

“Velocity.” This will be evaluated by the Brisk Walking Test. Participants will walk 30 m and the time will be recorded. Two reps with 1-min rest between them will be performed. The best trial will be used for computations.

“Functional reach.” The Functional Reach Test will be applied [59], in which participants will stand in front of a wall and must reach the maximum frontal distance with their arms raised to 90 degrees from the trunk, maintaining this position for a few seconds. The maximum distance reached perpendicularly to the wall will be recorded.

“Short physical performance battery (SPPB).” This battery will evaluate walking speed, balance, and time used to get up from a chair five times by direct observation [60].

“Self-perception of physical fitness.” For this evaluation the International Scale of Physical Fitness (IFIS) [61] will be used. Participants must answer different questions about their self-perception of general and cardio-respiratory fitness, strength, speed-agility and flexibility compared to their friends using a 5-level Likert scale. Answer options are: “very poor”, “poor”, “average”, “good”, and “very good”.

**Health-Related Quality of Life (HRQoL)**

EQ-5D-5L. This questionnaire evaluates the state of health, first in levels of severity by dimensions (descriptive system) and later through a visual analogical scale (VAS) [62]. The descriptive system contains five health dimensions (mobility, personal care, daily activities, pain/discomfort and anxiety/depression) and each one of them presents five severity levels (no problems, slight problems, moderate problems, serious problems and extreme problems/impossibility). Furthermore, participants should complete the VAS referencing the best state of health imaginable. The EQ-5D-5L questionnaire was developed from its preliminary Spanish version [62] proving to be valid and reliable.

15-D. This instrument has a total of 15 dimensions (mobility, vision, hearing, breathing, sleep, eating, speech, elimination, habitual activities, mental function, discomfort and symptoms, depression, anguish, vitality, and sexual activity) with 5 levels of response each, obtaining a single score ranging from 0 to 1, where 0 corresponds to the worst possible quality of life and 1 to the best [63,64]. The Spanish version of 15-D has been developed by the author of the questionnaire, with a rigorous process of translation and cultural adaptation. This version is available upon request from the author of the questionnaire; however, it has not been published.

**Depression**

To evaluate depressive symptoms, the Geriatric Depression Scale (GDS) questionnaire will be used. This scale consists of 15 questions about how the participant has felt in the last 14 days, with “yes” or “no” answers [65]. The GDS questionnaire has proven to be reliable and valid in the Spanish population [66].

**Happiness**

It will be evaluated through the Subjective Happiness Scale and the Satisfaction with Life Scale (SWLS).

This measure will be evaluated with the General Happiness Scale [67] which consists of 4 items where participants must respond between a score of 1 to 7, where 1 would imply less happiness and 7 more happiness. In addition, the Life Satisfaction Scale (SWLS) will also be used [68], which consists of 5 items using a 7-point Likert where 1 corresponds to strongly disagree and 7 to strongly agree. The Subjective Happiness Scale presented adequate reliability and validity in the Spanish population [69] has proven to be valid and reliable in the Spanish population [70].

**Physical activity and sedentary behaviors**

Physical activity in leisure time. It will be assessed through the Leisure Time Physical Activity Instrument (LTPAI). This questionnaire includes 4 items with 3 activity levels: light, moderate, and vigorous. Participants will be asked about the time per week that they had engaged in physical activity and at what activity level during the last 4 weeks. The scale will be simplified into three levels: (1) 0.5 to 1.5 h per week, (2) 2 to 4 h per week, and (3) more than 4 h per week. For the first two activity levels, the average number of hours (1 and 3 h, respectively) will be considered to calculate the total score. If no level is selected for a category, the number of hours will be equal to 0. Leisure-time physical activity level for one week will be obtained by the sum of the number of hours indicated by participants for every category of intensity [25].

**Physical Activity at Home and Work**

It will be measured using the Physical Activity at Home and Work (PAHWI) tool. It consists of seven items with 3 work levels performed at home (light, moderate, and heavy activity) and four levels for employment (sedentary, light, moderate, and heavy activity). The items present a brief explanation and participants should report on the time they spend on each. The result will be obtained by adding up the hours in each category to obtain the total score [25].

### 2.5. Statistical Analysis

Initially, to draw a clear profile of participants’ characteristics, descriptive analyses will be conducted. Data will be presented as Mean (M) and standard deviation (SD) for continuous variables and; as frequencies and percentages for categorical variables. Normality will be determined by the Kolmogorov–Smirnov test and Lilliefors correction). Between-group differences on participants’ characteristics will be analyzed using independent sample t-tests for continuous variables and Pearson’s chi square test will be performed for categorical data if data are normally distributed.

Between-groups differences will be checked applying the Mann–Whitney U test. Moreover, correlations between the main study variables, physical activity, fitness and HRQoL will be calculated using Pearson’s correlation coefficient with Bonferroni adjustment. Finally, a stepwise linear regression analysis will be carried out to comprehend which variables may influence fitness, physical activity, and participant’s HRQoL, including those parameters that show statistically significant associations with these parameters. Overall, statistical significance level will be set at *p* ≤ 0.05 for the most of test and regression. However, specifically for correlations, significance level will be fixed at *p* ≤ 0.005 due the number of comparisons.

### 2.6. Data Triangulation

**Figure 4:**
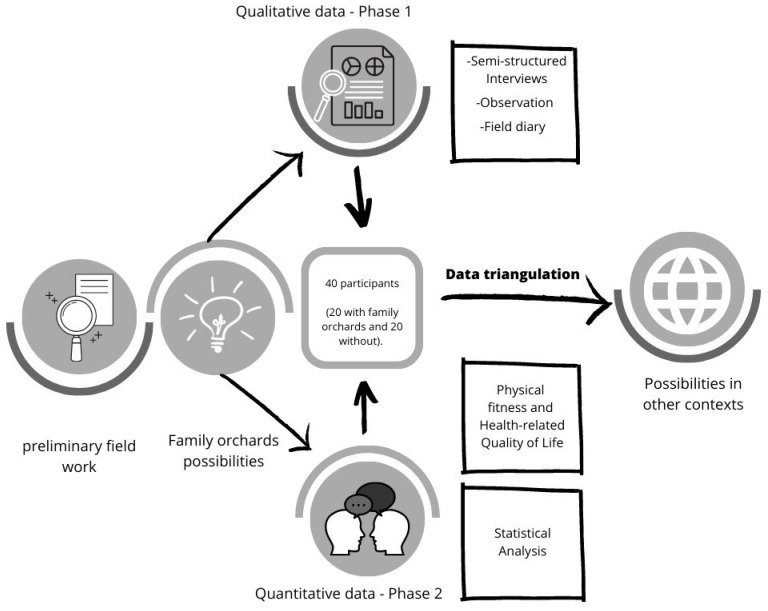
Final outline of proposed study protocol.

This procedure will be conducted by two members of the research team, (an anthropologist and a sports science specialist), who will examine, analyze and interpret all the materials obtained in the end, collaboration with a third expert in as a referee in case of discrepancies, thus completing the triangulation. The confluence of professionals with different origins, backgrounds, and skills has been proposed as an essential aspect to achieve success in an investigation such as the one proposed here. This interdisciplinary research allows the introduction of the anthropological view in the ways of thinking of specialists in physical activity. As opposed to the uniqueness of the laboratory, the diversity of the field and the concrete social life. Thus, the final outline of the study protocol is shown in Figure 4.

## 3. Discussion

Interdisciplinary studies have proved to be an excellent research strategy to address the complexity of the real world [71]. In fact, previous research has shown this trend [72]. Scientists with different disciplinary backgrounds often have different views on what count as good data, good evidence, a good model, or a good explanation [73]. With this research method, collaborations have emerged that were once unthinkable [74,75]. The social sciences in general and anthropology in particular have also contributed with their way of seeing the world. From ethnography it is possible to take into account social factors such as age, rurality index or gender [76]. Contributions to the field of health have been commonplace [77,78], and related to this is a growing collaboration with Sports Science professionals [79,80]. The present Study Protocol is a good example in this regard, identifying from field work the possible benefits of family orchards.

Family orchards have traditionally been understood as a piece of land that provides the population with several benefits, especially in rural contexts, as these natural spaces were essentially used to obtain food and represented the base of the local economy in rural areas [81]. In this respect, less economically developed rural areas in Extremadura (Spain) like Las Hurdes have survived during the last 50 years due to the work and efforts of the local population in these traditional orchards [12]. However, besides this economical support for the families, these orchards involve a wide variety of cultural, social, physical, and psychological processes that often are developed in relation to them [20,21].

Previous research has shown the beneficial effects of activities that are developed in nature such as gardening, farming or horticultural activities in older adults, since these activities contribute to improving physical health (such as, functional ability, greater physical activity, reduced weight gain and body mass index, decreased falls episodes, total cholesterol, blood pressure, etc.) [27,82,83]. Likewise, these activities contribute to an increase in well-being and quality of life [26,82], improve self-esteem and provide opportunities for social interaction [84] and psychological benefits, such as personal life satisfaction [33] and the reduction in depression and anxiety levels [85]. However, there is a lack of research regarding the effects on HRQOL and fitness in older people of a variety of similar activities also performed in nature such as tasks related with agriculture in family orchards, especially for those living in rural contexts. In this respect, despite traditional orchards having represented a way of living for the rural population throughout history, as previously stated, many other effects on their owners may arise from the work performed on these pieces of land.

This mixed study details the protocol and the methodology that will analyze the effects of working in family orchards on fitness status and health-related quality of life of elderly people in a rural population located in Las Hurdes (Spain). The assessment instruments that will be used in this study are standardized and validated into Spanish for the targeted population. The assessment procedures will be also adapted for participants, as this evaluation will be performed in the participants’ environment. In order to complement the outcomes and information obtained using the fitness tests previously described, other evaluation instruments will be employed such as semi-structured interviews and validated questionnaires. Thus, it is considered that the methodology of this study is robust and adequate to evaluate the effects and the importance of the family orchards for this population.

The outcomes of this study might enable us to design further physical exercise-based interventions using family orchards as an adequate resource to improve the health-related quality of life and fitness of older people in rural areas. Despite the relatively small sample size could present a limitation on the generalization of the results of fitness and HRQoL to other contexts. It is mainly caused by this being a depopulated area like most rural areas and implies an important handicap. Therefore, future studies in this context might require specific adjustments in this regard. However, physical activity to perform the basic and instrumental daily activities such as gardening, work in orchards or feeding livestock are culturally, and socially settled in rural environment, therefore, the study detailed here might also be applied to other similar rural areas in Spain and worldwide.

## 4. Conclusions

From a previous ethnography, we identified the possibility that family orchards were beneficial for the health of the elderly in rural areas. An interdisciplinary work team was then created to design this protocol which aims to analyze the effects of the use of these traditional family orchards as a daily resource on health-related quality of life, well-being, and fitness status in elderly from a rural environment of Las Hurdes (Spain). The findings of this mixed study will help—from the possibilities offered by interdisciplinary studies—to discover how the orchard activities influence health, well-being, and fitness, and if these activities cause a positive effect on population’s health-related quality of life. Thus, they could help to design different strategies and apply physical exercise-based interventions using family orchards as a main resource to improve the health-related quality of life, well-being, and fitness of older people in rural areas. Moreover, the possible developed strategies could be applied to other rural areas with similar characteristics.

## Figures and Tables

**Table 1:** Research techniques.

Research Technique	Empirical Material Obtained
Field Diary	Notes taken during fieldwork. Contextual data and other issues information
In-Depth Interviews	Semi-structured interviews, but designed to include additional content categories
Observational Units	Local people’s daily routine. Observation of type of physical activity carried out in the family orchards; Daily activities

## References

[1] European Commission (2015). The 2015 Ageing Report Economic and Budgetary Projections for the 28 EU Member States (2013–2060).

[2] World Health Organization (2015). World Report on Ageing and Health.

[3] United Nations (2015). World population ageing. Department of Economic and Social Affairs, Population 177 Division.

[4] García J.M. (2014). ¿Por qué vivimos más? Descomposición por causa de la esperanza de vida española de 1980 a 2009. Rev. Española Investig. Sociológicas.

[5] Van de Kaa D.J. The idea of a second demographic transition in industrialized countries. Proceedings of the Sixth Welfare Policy Seminar of the National Institute of Population and Social Security.

[6] Spanish National Statistic Institute (2019). Índices de Envejecimiento. En Indicadores de Estructura de la Población. https://www.ine.es/jaxiT3/Datos.htm?t=1418#!tabs-tabla.

[7] Spanish National Statistics Institute (INE) (2016). Population Projections 2016–2066. Madrid. http://www.ine.es/prensa/np994.pdf.

[8] Silvestre J. (2002). Las emigraciones interiores en España durante los siglos XIX y XX: Una revisión bibliográfica. Ager. Rev. Estud. Sobre Despoblación Desarro. Rural.

[9] Spanish National Statistic Institute (2020). Índices de envejecimiento por comunidad autónoma. En Indicadores de Estructura de la Población. https://www.ine.es/jaxiT3/Datos.htm?t=1452#!tabs-tabla.

[10] Legendre M. (1927). Las Jurdes: Étude de Géographie Humaine.

[11] Mateos J. (2017). La producción geosimbólica de Las Hurdes. Teoría, Historia y Práctica de un Territorio Imaginario. Ph.D. Thesis.

[12] Catani M. (1999). Las Hurdes como imagen de una sociedad local en transformación. Rev. Estud. Extrem..

[13] Hammersley M., Atkinson P. (1994). Etnografía: Métodos de Investigación.

[14] Galloza J., Castillo B., Micheo W. (2017). Benefits of Exercise in the Older Population. Phys. Med. Rehabilitation Clin. N. Am..

[15] Vagetti G.C., Filho V.C.B., Moreira N.B., De Oliveira V., Mazzardo O., De Campos W. (2014). Association between physical activity and quality of life in the elderly: A systematic review, 2000-2012. Rev. Bras. Psiquiatr..

[16] Nair P.K.R. (1993). An Introduction to Agroforestry.

[17] Gispert M., Vales A.M., Vilamajó D. (2010). Els horts familiars de Méxic i de Cuba. Interrelació existent entre l’entorn natural, la societat i les identitats culturals a l’Amèrica tropical. Rev. Ethnol. Catalunya.

[18] García J.C., Gutiérrez J., Balderas M.A., Araújo M.A. (2016). Estrategia de vida en el medio rural del Altiplano Central Mexicano: El huerto familiar. Agric. Soc. Desarro..

[19] Altieri M.A., Nicholls C.I. (2013). The adaptation and mitigation potential of traditional agriculture in a changing climate. Clim. Chang..

[20] García J., Gutiérrez C., Balderas M.Á., Araújo M.A. (2016). Sociocultural and environmental benefits from family orchards in the Central Highlands of México. Bois For. Trop..

[21] Rivas G. (2014). Huertos familiares para la conservación de la agrobiodiversidad, la promoción de la seguridad alimentaria y la adaptación al cambio climático. Ambientico.

[22] Catani M. (1998). Las Hurdes desde dentro y desde fuera. En II Congreso de Hurdanos y hurdanófilos.

[23] Calkins M., Szmerekovsky J.G., Biddle S. (2007). Effect of Increased Time Spent Outdoors on Individuals with Dementia Residing in Nursing Homes. J. Hous. Elder..

[24] Calvet-Mir L., Garnatje T., Parada M., Vallés J., Reyes-García V. (2016). Más allá de la producción de alimentos: Los huertos familiares como reservorios de diversidad biocultural. Ambienta.

[25] Munguía-Izquierdo D., Arrese A.L., Mannerkorpi K. (2011). Transcultural Adaptation and Psychometric Properties of a Spanish-Language Version of Physical Activity Instruments for Patients with Fibromyalgia. Arch. Phys. Med. Rehabil..

[26] Yao Y.-F., Chen K.-M. (2016). Effects of horticulture therapy on nursing home older adults in southern Taiwan. Qual. Life Res..

[27] Nicklett E.J., Anderson L.A., Yen I.H. (2016). Gardening Activities and Physical Health Among Older Adults. J. Appl. Gerontol..

[28] De Keijzer C., Tonne C., Sabia S., Basagana X., Valentín A., Singh-Manoux A., Antó J.M., Alonso J., Nieuwenhuijsen M.J., Sunyer J. (2019). Green and blue spaces and physical functioning in older adults: Longitudinal analyses of the Whitehall II study. Environ. Int..

[29] Braubach M., Egorov A., Mudu P., Wolf T., Thompson C.W., Martuzzi M., Kabisch N., Korn H., Stadler J., Bonn A. (2017). Effects of Urban Green Space on Environmental Health, Equity and Resilience BT—Nature-Based Solutions to Climate Change Adaptation in Urban Areas: Linkages between Science, Policy and Practice.

[30] Berg A.E.V.D., Van Winsum-Westra M., De Vries S., E Van Dillen S.M. (2010). Allotment gardening and health: A comparative survey among allotment gardeners and their neighbors without an allotment. Environ. Health.

[31] White M.P., Elliott L., Taylor T., Wheeler B.W., Spencer A., Bone A., Depledge M., Fleming L.E. (2016). Recreational physical activity in natural environments and implications for health: A population based cross-sectional study in England. Prev. Med..

[32] Largo-Wight E. (2011). Cultivating healthy places and communities: Evidenced-based nature contact recommendations. Int. J. Environ. Health Res..

[33] Maas J., Verheij R.A., Groenewegen P.P., de Vries S., Spreeuwenberg P. (2006). Green space, urbanity, and health: How strong is the relation?. J. Epidemiol. Community Health.

[34] Mejias A.I. (2014). Contribution of urban vegetable gardens to health. Hábitat Soc..

[35] Pecurul M., Cristóbal R., & Moscoso D.J. (2006). La contribución de los espacios verdes y los bosques a la mejora de la salud y al bienestar. Ambienta.

[36] Bellows A.C., Brown K., Smit J. (2003). Health Benefits of Urban Agriculture.

[37] Wakefield S., Yeudall F., Taron C., Reynolds J., Skinner A. (2007). Growing urban health: Community gardening in South-East Toronto. Health Promot. Int..

[38] Andreucci M.B., Russo A., Olszewska-Guizzo A. (2019). Designing Urban Green Blue Infrastructure for Mental Health and Elderly Wellbeing. Sustainability.

[39] Hu X., Jing M., Zhang M., Yang P., Yan X. (2020). Responsiveness and minimal clinically important difference of the EQ-5D-5L in cervical intraepithelial neoplasia: A longitudinal study. Heal. Qual. Life Outcomes.

[40] Garcia-Gordillo M.Á., Adsuar J.C., Olivares P.R. (2015). Normative values of EQ-5D-5L: In a Spanish representative population sample from Spanish Health Survey, 2011. Qual. Life Res..

[41] Creswell J.W., Poth C.N. (2016). Qualitative Inquiry and Research Design: Choosing Among Five Approaches.

[42] Wiley J. (2016). Mini-mental state examination (MMSE) para la detección de demencia en personas mayores de 65 años o mayores sin evaluación clínica en poblaciones de atención primaria de la comunidad. Rev. Médica Clínica Las Condes.

[43] Cantero-Garlito P., Moruno-Miralles P., Flores-Martos J.A. (2020). Mothers Who Take Care of Children with Disabilities in Rural Areas of a Spanish Region. Int. J. Environ. Res. Public Health.

[44] Rivero-Jiménez B., Conde-Caballero D., Mariano-Juárez L. (2020). Health and Nutritional Beliefs and Practices among Rural Elderly Population: An Ethnographic Study in Western Spain. Int. J. Environ. Res. Public Health.

[45] Lincoln Y.S., Denzin N.K. (2000). Handbook of Qualitative Research.

[46] Rose D.J., Jones C.J., Lucchese N. (2002). Predicting the Probability of Falls in Community-Residing Older Adults Using the 8-Foot Up-and-Go: A New Measure of Functional Mobility. J. Aging Phys. Act..

[47] Spagnuolo D.L., Jürgensen S.P., Iwama A.M., Dourado V.Z. (2010). Walking for the Assessment of Balance in Healthy Subjects Older than 40 Years. Gerontology.

[48] Evans T., Jefferson A., Byrnes M., Walters S., Ghosh S., Mastaglia F.L., Power B., Anderton R.S. (2017). Extended “Timed Up and Go” assessment as a clinical indicator of cognitive state in Parkinson’s disease. J. Neurol. Sci..

[49] Springer B.A., Marin R., Cyhan T., Roberts H., Gill N.W. (2007). Normative Values for the Unipedal Stance Test with Eyes Open and Closed. J. Geriatr. Phys. Ther..

[50] Vellas B.J., Ms S.J.W., Romero L., Baumgartner R.N., Rubenstein L.Z., Garry P.J. (1997). One-Leg Balance Is an Important Predictor of Injurious Falls in Older Persons. J. Am. Geriatr. Soc..

[51] Yardley L., Beyer N., Hauer K., Kempen G., Piot-Ziegler C., Todd C. (2005). Development and initial validation of the Falls Efficacy Scale-International (FES-I). Age Ageing.

[52] Kempen G.I.J.M., Todd C.J., Van Haastregt J.C.M., Zijlstra G.A.R., Beyer N., Freiberger E., Hauer K.A., Piot-Ziegler C., Yardley L. (2007). Cross-cultural validation of the Falls Efficacy Scale International (FES-I) in older people: Results from Germany, the Netherlands and the UK were satisfactory. Disabil. Rehabil..

[53] Lomas-Vega R., Hita-Contreras F., Mendoza N., Martínez-Amat A. (2012). Cross-cultural adaptation and validation of the Falls Efficacy Scale International in Spanish postmenopausal women. Menopause.

[54] Scheffer A.C., Schuurmans M.J., Vandijk N., Van Der Hooft T., E De Rooij S. (2010). Reliability and validity of the visual analogue scale for fear of falling in older persons. J. Am. Geriatr. Soc..

[55] Rikli R.E., Jones C.J. (1999). Development and Validation of a Functional Fitness Test for Community-Residing Older Adults. J. Aging Phys. Act..

[56] Ruiz-Ruiz J., Mesa J.L., Gutiérrez A., Castillo M.J. (2002). Hand size influences optimal grip span in women but not in men. J. Hand Surg..

[57] Ito T., Shirado O., Suzuki H., Takahashi M., Kaneda K., Strax T.E. (1996). Lumbar trunk muscle endurance testing: An inexpensive alternative to a machine for evaluation. Arch. Phys. Med. Rehabil..

[58] Jones C.J., Rikli R.E., Max J., Noffal G. (1998). The Reliability and Validity of a Chair Sit-and-Reach Test as a Measure of Hamstring Flexibility in Older Adults. Res. Q. Exerc. Sport.

[59] Duncan P.W., Weiner D.K., Chandler J., Studenski S. (1990). Functional Reach: A New Clinical Measure of Balance. J. Gerontol..

[60] Guralnik J.M., Simonsick E.M., Ferrucci L., Glynn R.J., Berkman L.F., Blazer D.G., Scherr P.A., Wallace R.B. (1994). A Short Physical Performance Battery Assessing Lower Extremity Function: Association with Self-Reported Disability and Prediction of Mortality and Nursing Home Admission. J. Gerontol..

[61] Álvarez-Gallardo I.C., Soriano-Maldonado A., Segura-Jiménez V., Carbonell-Baeza A., Estévez-López F., McVeigh J.G., Delgado-Fernández M., Ortega F.B. (2016). International Fitness Scale (IFIS): Construct Validity and Reliability in Women with Fibromyalgia: The al-Ándalus Project. Arch. Phys. Med. Rehabil..

[62] Herdman M., Gudex C., Lloyd A., Janssen M., Kind P., E Parkin D., Bonsel G.J., Badia X. (2011). Development and preliminary testing of the new five-level version of EQ-5D (EQ-5D-5L). Qual. Life Res..

[63] Laas K., Roine R., Räsänen P., Sintonen H., Leirisalo-Repo M. (2008). HUS QoL Study Group Health-related quality of life in patients with common rheumatic diseases referred to a university clinic. Rheumatol. Int..

[64] Sintonen H. (2001). The 15D instrument of health-related quality of life: Properties and applications. Ann. Med..

[65] Yesavage J.A., Brink T., Rose T.L., Lum O., Huang V., Adey M., Leirer V.O. (1982). Development and validation of a geriatric depression screening scale: A preliminary report. J. Psychiatr. Res..

[66] Martín M.I.F.S., Andrade-Rosa C., Molina J.D., Muñoz P.E., Carretero B., Rodríguez M., Silva A. (2002). Validation of the Spanish version of the geriatric depression scale (GDS) in primary care. Int. J. Geriatr. Psychiatry.

[67] Lyubomirsky S., Lepper H.S. (1999). A Measure of Subjective Happiness: Preliminary Reliability and Construct Validation. Soc. Indic. Res..

[68] Diener E., Emmons R.A., Larsen R.J., Griffin S. (1985). The Satisfaction with Life Scale. J. Pers. Assess..

[69] Extremera N., Salguero J.M., Fernández-Berrocal P., Salguero J.M. (2010). Trait Meta-Mood and Subjective Happiness: A 7-week Prospective Study. J. Happiness Stud..

[70] Vazquez C., Duque A., Hervás G. (2013). Satisfaction with Life Scale in a Representative Sample of Spanish Adults: Validation and Normative Data. Span. J. Psychol..

[71] Danermark B. (2019). Applied interdisciplinary research: A critical realist perspective. J. Crit. Realism.

[72] Klein J.T. (2008). Evaluation of Interdisciplinary and Transdisciplinary Research. Am. J. Prev. Med..

[73] Green S., Andersen H. (2019). Systems science and the art of interdisciplinary integration. Syst. Res. Behav. Sci..

[74] Howes L.M. (2015). Developing the Methodology for an Applied, Interdisciplinary Research Project: Documenting the Journey Toward Philosophical Clarity. J. Mix. Methods Res..

[75] Susman E.J., Marceau K., Dockray S., Ram N. (2019). Interdisciplinary Work Is Essential for Research on Puberty: Complexity and Dynamism in Action. J. Res. Adolesc..

[76] Rivero-Jiménez B., Conde-Caballero D., González B.M., Juarez L.M. (2020). Technology to Improve Elderly Nutrition. Advances in Medical Technologies and Clinical Practice.

[77] Rosso A. (2015). Conversations between anthropology and psychiatry: Drawing out the best from interdisciplinarity in global mental health. Australas. Psychiatry.

[78] Singer M. (2009). Interdisciplinarity and collaboration in responding to HIV and AIDS in Africa: Anthropological perspectives. Afr. J. AIDS Res..

[79] Ford A. (2019). Sport horse leisure and the phenomenology of interspecies embodiment. Leis. Stud..

[80] Smith B.M., Caddick N. (2012). Qualitative methods in sport: A concise overview for guiding social scientific sport research. Asia Pac. J. Sport Soc. Sci..

[81] Moyano E. (2014). La agricultura familiar revisitada. Una mirada a la agricultura como factor de desarrollo social y económico. Ambienta.

[82] Strout K., Jemison J., O’Brien L., Wihry D., Waterman T. (2017). GROW: Green Organic Vegetable Gardens to Promote Older Adult Wellness: A Feasibility Study. J. Community Heal. Nurs..

[83] Walsh J.M.E., Pressman A.R., Cauley J.A., Browner W.S. (2001). Predictors of physical activity in community-dwelling elderly white women. J. Gen. Intern. Med..

[84] Wang D., Glicksman A. (2013). “Being Grounded”: Benefits of Gardening for Older Adults in Low-Income Housing. J. Hous. Elder..

[85] Soga M., Gaston K., Yamaura Y. (2017). Gardening is beneficial for health: A meta-analysis. Prev. Med..

